# Refractory eczematous dermatitis arising after allogeneic hematopoietic stem cell transplantation responsive to dupilumab

**DOI:** 10.1016/j.jdcr.2024.08.009

**Published:** 2024-08-30

**Authors:** Jenny Lai, Eleanor Russell-Goldman, Connie R. Shi

**Affiliations:** a Harvard Medical School, Boston, Massachusetts; b Department of Dermatology, Brigham and Women’s Hospital, Boston, Massachusetts; c Department of Pathology, Brigham and Women’s Hospital, Boston, Massachusetts; d Center for Cutaneous Oncology, Dana-Farber Cancer Institute, Boston, Massachusetts

**Keywords:** bone marrow transplant, cutaneous toxicity, dupilumab, eczematous dermatitis, eczematous graft-versus-host disease, oncodermatology

## Introduction

Eczematous dermatitis has been reported to arise after allogeneic hematopoietic stem cell transplantation (HSCT), presenting as either de novo eczematous dermatitis,^1^ postulated transfer of atopic predisposition from donor to recipient,^2^ or as an eczematous subtype of graft-versus-host disease (GVHD).^3^ There are recent reports of pediatric patients with eczematous GVHD following HSCT that responded to dupilumab.^4^, ^5^, ^6^, ^7^ We report a case of an adult patient with a history of allogeneic HSCT and no prior history of atopy or eczema who developed an eczematous dermatitis refractory to first and second line GVHD treatment with oral corticosteroids and ruxolitinib and was successfully treated with dupilumab.

## Case report

A 19-year-old female with a past medical history of aplastic anemia underwent an 8/8 matched unrelated donor allogeneic HSCT with bone marrow source (fully matched at human leukocyte antigen (HLA)-A, B, C, and DRB1 locus; 9/10 with one HLA-DQB1 mismatch; sex mismatch was also present). Her reduced intensity conditioning regimen included cyclophosphamide, fludarabine, rabbit antithymocyte globulin, and total body irradiation. GVHD prophylaxis included oral tacrolimus and methotrexate (10 mg/m^2^ on post-transplant days +1, 3, 6, and 11). She had no personal or family history of atopic dermatitis (AD). Donor history for AD was unknown.

**Fig 1:**
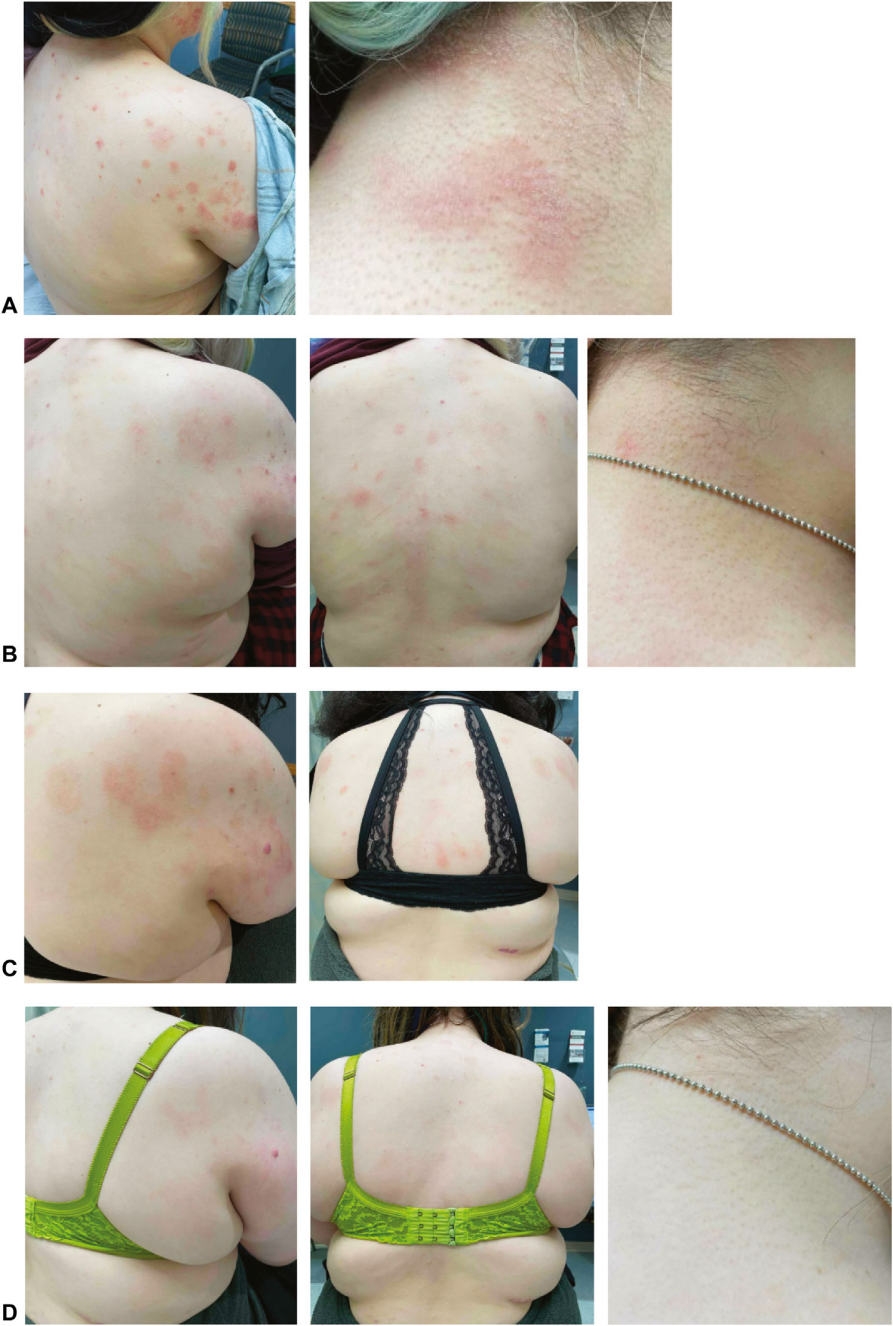
Eczematous dermatitis arising after allogenic HSCT in a 19-year-old female who presented with nummular erythematous scaly patches and plaques on the neck, trunk, and extremities. **A,** Skin exam on day 239 post-transplant despite treatment with oral prednisone, ruxolitinib, and tacrolimus in addition to topical corticosteroids and tacrolimus. A skin biopsy was performed from the right upper arm. **B,** Improvement in rash 1 month after dupilumab initiation. **C,** Three months after dupilumab initiation. **D,** No active rash after 6 months of dupilumab. *HSCT*, Hematopoietic stem cell transplantation.

**Fig 2:**
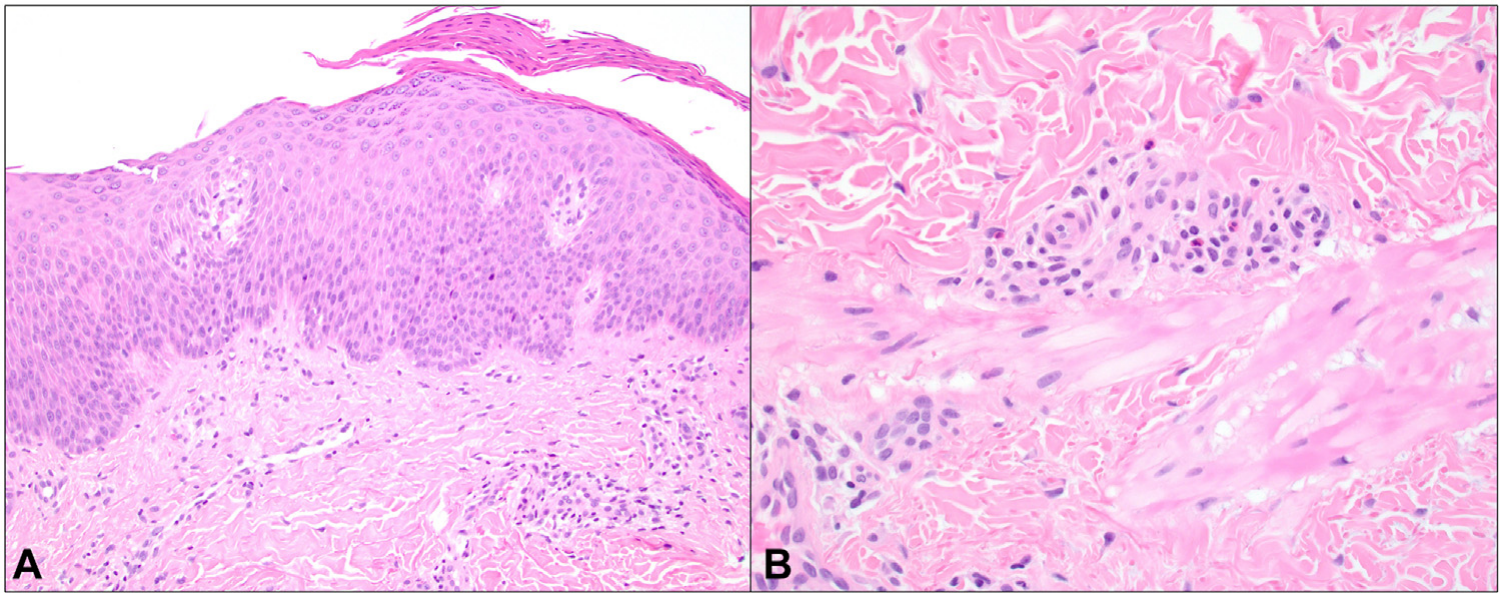
Histopathologic features supporting an eczematous dermatitis. **A,** Histopathologic examination showed a subacute spongiotic dermatitis with parakeratosis and a relatively sparse superficial dermal chronic inflammatory infiltrate. There was no evidence of an interface component or satellitosis (200× magnification). **B,** Easily identifiable eosinophils were also present, which would be a less common finding in graft-versus-host disease (400× magnification).

On day 152 post-transplantation, she developed diffuse pruritus without a rash. Prior to this, the patient had no manifestations of either acute or chronic GVHD. She was empirically treated with 0.5 mg/kg prednisone for presumed GVHD and continued tacrolimus 1 mg twice daily for GVHD prophylaxis. A few weeks later, she developed nummular eczematous patches on the trunk. The clinical differential included eczematous GVHD given her history of allogeneic HSCT versus eczematous dermatitis. Despite ongoing treatment with prednisone, clobetasol 0.05% ointment, tacrolimus 0.1% ointment, and the addition of ruxolitinib 5 mg twice daily for presumed eczematous GVHD, the patient developed worsening nummular eczematous patches on her trunk and extremities with lichenification and crusting, including involvement of the flexor elbows classically associated with AD. She did not have any extracutaneous organ system involvement of GVHD (ocular, oral, gastrointestinal, liver, pulmonary, musculoskeletal, or genital). Complete blood count was unremarkable and showed a normal absolute eosinophil count throughout the entire course of the rash. She did not develop any food or environmental allergies in tandem with the dermatitis. By day 239 post-transplantation, she had persistent eczematous dermatitis with 10/10 pruritus on the trunk and extremities (Fig 1, *A*) involving approximately 10% body surface area and consistent with investigator’s global assessment for AD score of 3. A skin punch biopsy demonstrated subacute spongiotic dermatitis with superficial perivascular lymphocytic infiltrate and eosinophils (Fig 2). Of note, typical features of GVHD such as an interface component, satellitosis, lichenoid inflammation, and sclerosis were not seen. Given the persistence of the rash despite systemic treatment with oral prednisone and ruxolitinib for GVHD, ongoing GVHD prophylaxis with tacrolimus, as well as topical corticosteroids and topical tacrolimus, she was initiated on dupilumab 300 mg every other week. After 1 month, the rash improved significantly with itch severity improving to 3/10 (Fig 1, *B*). Oral tacrolimus was discontinued and prednisone was tapered. After 3 months on dupilumab, she reported complete resolution of itch, continued improvement in the rash, and no adverse effects from dupilumab (Fig 1, *C*). Due to continued improvement, prednisone was discontinued. After 6 months on dupilumab, she had complete skin clearance (body surface area 0%, investigator’s global assessment score 0), and itch remained completely resolved (Fig 1, *D*). She was successfully tapered off all systemic immunosuppressants, did not require use of topical corticosteroids or tacrolimus, and continued dupilumab 300 mg every other week.

## Discussion

Eczematous dermatitis arising in patients who have undergone allogeneic HSCT can pose a diagnostic and treatment dilemma, with a differential that includes de novo eczematous dermatitis, potential transfer of atopic predisposition from donor to host, or eczematous GVHD. There are reports of eczematous dermatitis developing in patients without history of atopy after unrelated cord blood transplantation, HLA-matched related donor transplantation, and HLA-matched unrelated donor transplantation with a clinical course that differs from classic GVHD and more closely resembles AD, including involvement of flexural areas and a chronic and relapsing course.^1^ In this cohort, patients most often responded to topical steroids, topical calcineurin inhibitors, and occasionally phototherapy, with infrequent need for systemic immunosuppression.^1^ Eczematous GVHD, on the other hand, is a subtype of GVHD that can be refractory to conventional therapy.^3^^,^^5^^,^^6^ Histopathology demonstrates spongiosis, acanthosis, and lymphocytic and eosinophilic infiltrates^3^ often with concurrent changes of vacuolar interface reaction and apoptotic keratinocytes characteristic of GVHD.^8^

This report illustrates the complete response of new-onset eczematous dermatitis to dupilumab in an adult patient with a history of allogeneic HSCT who was refractory to systemic immunosuppressants such as prednisone and ruxolitinib, suggesting that dupilumab is an effective, safe, and well-tolerated treatment for this subtype of dermatitis in adult allogeneic HSCT recipients. The patient did not meet diagnostic criteria for chronic cutaneous GVHD according to the National Institutes of Health (NIH) Consensus criteria,^9^ had no other GVHD-related organ system involvement, and histopathology did not demonstrate features of chronic GVHD, therefore making this case insufficient to meet criteria for an eczematous subtype of chronic GVHD. There are emerging reports of recalcitrant pediatric cases of eczematous GVHD successfully treated with dupilumab in children with a history of unrelated cord blood transplants.^4^, ^5^, ^6^ However, some of these cases reported as eczematous GVHD did not formally comment on NIH Consensus criteria for GVHD, did not have extracutaneous GVHD-related organ involvement, did not have a biopsy, or had biopsies demonstrating eczematous histopathology without typical features of GVHD.^5^ Our case highlights the importance of considering the NIH Consensus criteria in the evaluation of HSCT patients who present with cutaneous eruptions with GVHD in the differential.

Benefits of dupilumab in this case included complete clearance of an eczematous dermatitis that was refractory to ruxolitinib and prednisone, resolution of pruritus, and successful discontinuation of all systemic immunosuppressants without any flares. There are prior reported pediatric cases of eczematous GVHD that did not respond to ruxolitinib and improved after several doses of dupilumab.^4^^,^^5^^,^^7^ Similar to our patient, 2 of these pediatric cases had biopsies showing mild spongiosis without features of GVHD, consistent with an eczematous dermatitis.^4^^,^^5^ In line with these cases, our patient’s itch responded to the first dose of dupilumab, in contrast to the lack of improvement with months of prednisone and ruxolitinib. This case highlights that dupilumab can successfully treat refractory eczematous dermatitis arising post-HSCT in adults as well. Since ruxolitinib is a selective inhibitor of janus kinase 1 and 2, lack of response to ruxolitinib in these cases may be due to activation of other kinases that are more broadly inhibited by dupilumab. Further investigation is required to delineate differences in eczematous dermatitis arising after HSCT that may underlie differential response to dupilumab versus ruxolitinib.

Dupilumab blocks the signaling of type 2 helper T cell (Th2) cytokines interleukin-4 and interleukin-13.^4^ Th2 cells are elevated in eczematous GVHD and eczematous dermatitis, indicating a role of hyperactive Th2 immune responses in the pathophysiology.^10^ A further benefit of dupilumab in treating eczematous GVHD or eczematous dermatitis in this patient cohort is the avoidance of further systemic immunosuppression. Future studies in larger controlled cohorts should evaluate dupilumab as a potential first-line systemic therapy for skin-limited eczematous GVHD and eczematous dermatitis arising in allogeneic HSCT recipients.

## Conflicts of interest

Dr Shi reports conflicts with VisualDX (honoraria/consulting), unrelated to the scope of this work. Drs Russell-Goldman and Lai have no conflicts of interest to declare.
